# Proteostasis disruption and lipid dyshomeostasis in neurodegeneration: exploring common druggable targets across sporadic and monogenic disorders

**DOI:** 10.3389/fnmol.2025.1681079

**Published:** 2025-10-24

**Authors:** Priscila Pereira Sena, Lea Friedrich, Alcibiades Villarreal, Florian Fath, Liubovi Sopco, Mar Hernández-Guillamon, Maria Luiza Saraiva-Pereira, Gabrielle Britton, Jonasz Jeremiasz Weber, Thorsten Schmidt

**Affiliations:** ^1^Institute of Medical Genetics and Applied Genomics, Eberhard Karls University Tübingen, Tübingen, Germany; ^2^Instituto de Investigaciones Científicas y Servicios de Alta Tecnología (INDICASAT-AIP), Ciudad del Saber, Panama City, Panama; ^3^Sistema Nacional de Investigación (SNI) in Panama, Secretaría Nacional de Ciencia Tecnología e Innovación (SENACYT), Panama City, Panama; ^4^Department of Human Genetics, Ruhr University Bochum, Bochum, Germany; ^5^Department of Neurology, Huntington-Center NRW, St. Josef-Hospital Bochum, Ruhr University Bochum, Bochum, Germany; ^6^Neurovascular Research Laboratory, Vall d'Hebron Research Institute (VHIR), Barcelona, Spain; ^7^Serviço de Genética Médica, Hospital de Clínicas de Porto Alegre, Porto Alegre, Brazil; ^8^Departamento de Bioquímica, Universidade Federal do Rio Grande do Sul, Porto Alegre, Brazil; ^9^Centro de Vacunación e Investigación (CEVAXIN), Panama City, Panama

**Keywords:** apolipoprotein E, ApoE, aggregates, autophagy, amyloid β, cholesterol, plaques, polyglutamine

## Abstract

Neurodegenerative disorders pose an increasing burden in the aging society. These conditions share several molecular pathomechanisms, some of which may offer opportunities for therapeutic intervention. In this review, we explore a representative selection of sporadic and hereditary neurodegenerative diseases—namely Alzheimer's disease, cerebral amyloid angiopathy, and the polyQ disorders spinocerebellar ataxia types 2 and 3, as well as Huntington's disease—which all feature the accumulation of intra- or extracellular protein deposits as a hallmark. We place particular emphasis on dysregulations in proteostasis—underlying the formation of these aggregates—and the less commonly addressed disturbances in lipid metabolism. By highlighting potential mechanistic links across different classes of neurodegenerative diseases, we aim to provide new insights that may guide the identification of shared druggable targets and the development of broad-spectrum therapeutic strategies.

## 1 Introduction

With increasing life expectancy, the heterogeneous group of neurodegenerative disorders presents a significant and growing challenge to healthcare systems worldwide. Despite decades of intensive research, effective treatment options remain limited, underscoring the need for a deeper understanding of the diverse molecular mechanisms that ultimately lead to irreversible neuronal damage and death. This is likely due to the high heterogenicity of such conditions, which encompass both sporadic forms, such as most manifestations of Alzheimer's Disease (AD), Amyotrophic Lateral Sclerosis (ALS) or Parkinson's Disease (PD), and monogenic forms such as the hereditary triplet repeat disorders, including the polyglutamine (polyQ) diseases. These conditions vary in their age of onset, affected tissues, clinical manifestations, and underlying molecular pathways (Wilson et al., 2023; Kelser et al., 2024). For instance, while AD primarily affects memory and cognition through cortical and hippocampal pathology, PD is characterized by motor symptoms driven by dopaminergic neurodegeneration in the substantia nigra, and spinocerebellar ataxias (SCAs) predominantly impair motor circuits through cerebellar degeneration (Knopman et al., 2021; Poewe et al., 2017; Klockgether et al., 2019). This clinical and pathological diversity complicates diagnosis, treatment, and the development of broadly effective therapies.

Current treatment options remain largely symptomatic and disease-specific. In AD, acetylcholinesterase inhibitors and the NMDA receptor antagonist memantine can provide modest symptomatic relief but do not alter disease progression (Zhang et al., 2024). PD management relies heavily on dopaminergic replacement therapies such as levodopa, which improve motor symptoms but often lose effectiveness over time and do not halt neurodegeneration (Charvin et al., 2018). In ALS, drugs such as riluzole and edaravone extend survival only modestly (Jaiswal, 2019). For hereditary polyQ disorders like Huntington's disease (HD) and SCAs, unfortunately, no approved disease-modifying treatments exist to date (Tenchov et al., 2024). Collectively, these limitations highlight the urgent need for therapies that address the root causes of neurodegeneration.

At the molecular level, several pathogenic processes have been identified, including dysfunctional proteostasis leading to protein aggregation, mitochondrial and synaptic dysfunction, oxidative stress, neuroinflammation, and disturbances in lipid metabolism (Knopman et al., 2021; Poewe et al., 2017; Klockgether et al., 2019). While these mechanisms are well-studied individually, a major gap remains in understanding how they converge and interact across different neurodegenerative conditions.

In this review, we examine a selection of neurodegenerative disorders encompassing both sporadic and monogenic forms with proteopathic characteristics. By focusing on shared molecular features—particularly dysfunctional proteostasis as the driver of the hallmark protein aggregation—we draw attention to dysregulated lipid metabolism as a common contributor for this impairment. Through this perspective, we aim to support ongoing efforts toward the development of unifying therapeutic strategies capable of targeting multiple neurodegenerative conditions.

## 2 Neurodegenerative disorders

Neurodegenerative disorders are caused by progressive neuronal loss across multiple brain regions. With variable clinical and pathological presentations, this group consists of largely sporadic disorders such as Alzheimer's and Parkinson's disease (AD and PD, respectively) (Bali et al., 2012; Schulze et al., 2018) and other inherited diseases such as the polyglutamine disorders, which result from constitutional mutations of single genes, thus named monogenic hereditary disorders (Pihlstrøm et al., 2017). One of the most common hallmarks of neurodegenerative disorders is the aggregation of misfolded proteins into insoluble inclusion bodies within the nucleus or cytoplasm, such as Lewy bodies in PD, neurofibrillary tangles in AD, or polyQ aggregates in HD, as well as the formation of extracellular deposits in neuronal tissue, including neuritic amyloid plaques in AD (Klockgether et al., 2019; Knopman et al., 2021; Ross and Poirier, 2004). These deposits are a consequence of disruptions in a process collectively known as proteostasis—an intricate network of mechanisms that regulate protein synthesis, folding, trafficking, and degradation to maintain cellular protein homeostasis. Numerous molecular pathways involved in proteostasis have been identified and characterized in the context of neurodegeneration, either contributing to disease pathogenesis or acting as disease modifiers (Yerbury et al., 2016).

One potential, yet not fully understood, contributor is lipid metabolism, which is essential for both neuronal and glial function. When disturbed, it increases the risk of neurological disease, as strikingly demonstrated by the association of the apolipoprotein E (ApoE) allele ε4 (*APOE* ε4) with late-onset AD (Strittmatter et al., 1993; Kawade and Yamanaka, 2024; Yang et al., 2023). Although a direct link between protein aggregation and lipid metabolism in neurodegeneration may not be immediately apparent, emerging intersections suggest a relevant interplay (Hernandez-Diaz and Soukup, 2020), which will be explored in the following sections.

### 2.1 Sporadic neurodegenerative disorders

The vast majority of neurodegenerative disorders have a sporadic etiology, with only about 10% of cases considered hereditary (Dilliott et al., 2021). Non-genetic components—such as lifestyle and environmental factors—have been reported to either contribute to or protect against the development of common neurodegenerative conditions in the elderly, including AD, PD, and cerebral amyloid angiopathy (CAA) (Jäkel et al., 2022; Mentis et al., 2021).

Given the well-established role of lipid metabolism and ApoE in neurodegenerative dementias, we focus on AD and CAA as prototypical examples of predominantly sporadic neurodegenerative disorders, which are further characterized by the deposition of amyloidogenic proteins.

#### 2.1.1 Alzheimer's disease (AD)

**Figure 1:**
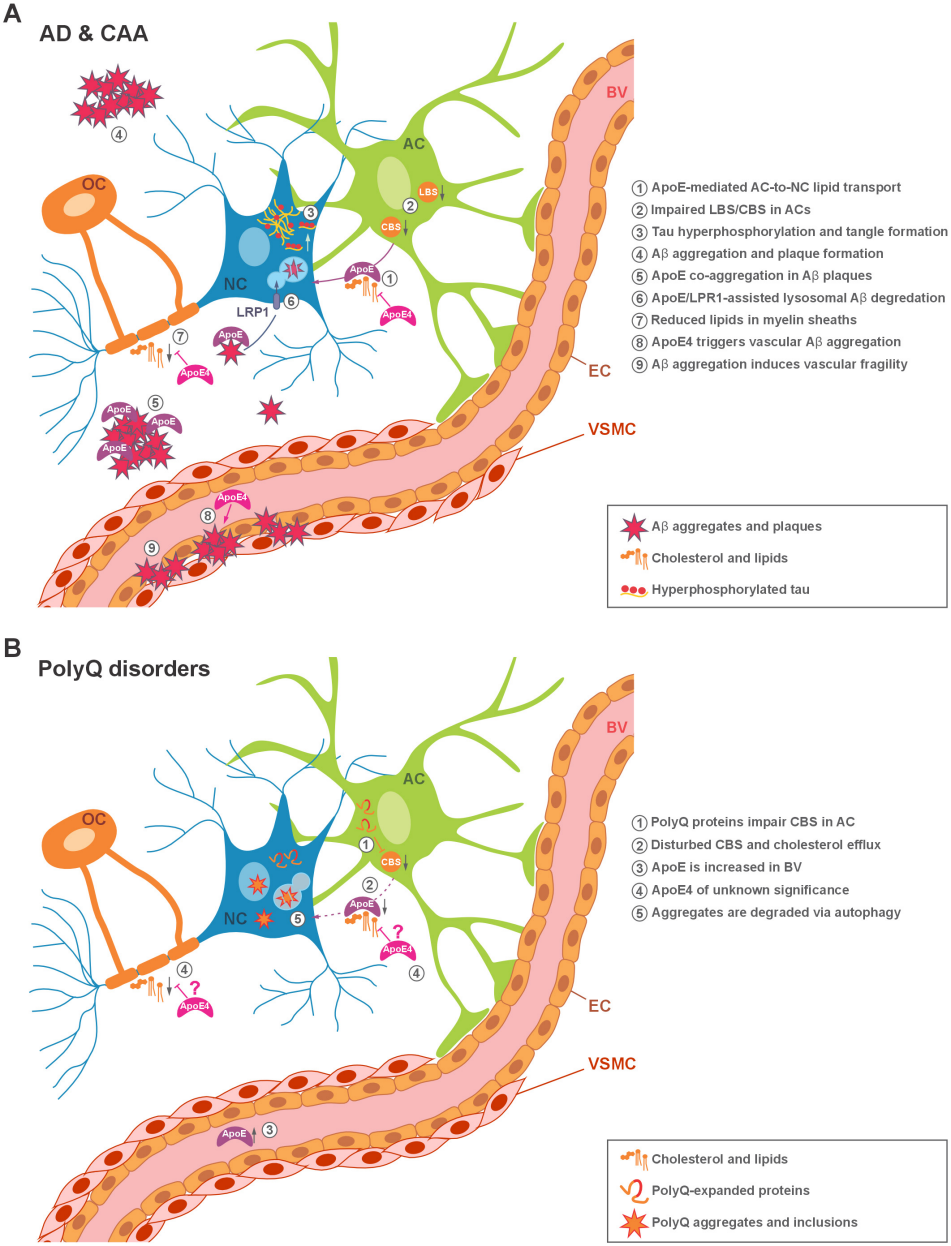
Illustration of pathogenic protein aggregate sites in the monogenic and sporadic neurodegenerative disorders covered in this review, and the known contribution of lipid metabolism and ApoE in the respective disorders. **(A)** In Alzheimer's disease (AD), neurofibrillary tangles composed of hyperphosphorylated tau accumulate within neurons, while Aβ deposits in the form of amyloid plaques are found in the extracellular space, where activated astrocytes may contribute to their uptake and removal. In cerebral amyloid angiopathy (CAA), amyloid plaques accumulate in the walls of blood vessels. **(B)** In Huntington's disease (HD) and spinocerebellar ataxias (SCAs) types 2 and 3, the respective disease-associated polyglutamine (polyQ)-expanded proteins form intracellular aggregates in the cytoplasm or nucleus of affected neurons. Characteristic events are numbered and described in the respective panel. AC, astrocyte; NC, neuronal cell; OC, oligodendrocyte; LBS, lipid biosynthesis; CBS, cholesterol biosynthesis; BV, blood vessel; EC, endothelial cells; VSMC, vascular smooth muscle cells; LRP1, lipoprotein receptor-related protein 1.

AD, the most common form of dementia, is characterized by cognitive impairment and neuronal loss that progress through several stages, each defined by distinct pathological and clinical features. AD is estimated to affect around 130 million individuals by 2050 if no therapies become available (Ju and Tam, 2022). A histopathological hallmark of AD is the accumulation of abnormal protein aggregates, particularly intracellular neurofibrillary tangles formed by hyperphosphorylated tau and extracellular amyloid-β (Aβ) plaques—composed of Aβ peptides generated by proteolytic cleavage of the amyloid precursor protein—in the limbic and neocortical regions (Chen et al., 2017; Zhao and Huai, 2023) (Figure 1). The microtubule-stabilizing protein tau exists in six isoforms generated by alternative splicing of the *MAPT* gene. Under physiological conditions, the ratio of three-repeat (3R) to four-repeat (4R) tau isoforms is tightly regulated, but this balance becomes disrupted in tau-related neurodegenerative disorders, altering tau's phosphorylation status and aggregation propensity (Rawat et al., 2022). Hyperphosphorylation causes tau to dissociate from microtubules, after which it aggregates into tangles with neurotoxic properties. Among the 85 identified phosphorylation sites, threonine 217 has emerged as a particularly important biomarker in AD. Phosphorylation at this residue produces p-tau217, whose elevated plasma levels are associated with early stages of the disease and correlate with cognitive decline. These findings suggest that p-tau217 not only reflects underlying tau pathology but also offers promise for early diagnosis and monitoring of disease progression (Hirota et al., 2025; Martin et al., 2013). The impairment of Aβ clearance mechanisms seems to be a major contributor to the accumulation of Aβ and tau in the brain, thus reflecting a failure of the cellular machinery responsible for protein quality control (Mawuenyega et al., 2010). The development of these plaques and tangles can also be a result of an imbalance between the production and degradation of proteins (He et al., 2020).

In neurons, whose functionality greatly depends on an exactly maintained proteome, disruption of proteostasis and accumulation of toxic aggregates is particularly detrimental, as it impairs crucial cellular functions and renders neurons vulnerable to stressors such as oxidative stress, chronic inflammation, and endogenous neurotoxins (e.g., quinolinic acid), which can precipitate neurodegeneration. Over the past decade, research has increasingly focused on elucidating the molecular consequences of proteostasis disturbances in AD. For example, impairment of key intracellular mechanisms such as the ubiquitin-proteasome system (UPS) reduces the degradation of misfolded proteins, notably Aβ and tau, as evidenced by decreased proteasomal complex subunits and defective nuclear localization of Nrf1, which normally promotes proteasome gene expression. Concurrently, molecular chaperones show diminished activity, further compromising proper protein folding and facilitating aggregation (Batko et al., 2024). Together, these molecular failures exacerbate proteotoxic stress, contributing to a vicious cycle of neuronal dysfunction and degeneration, and highlighting potential therapeutic targets aimed at restoring UPS activity and chaperone function.

Aging is a major risk factor for AD and is closely associated with a decline in proteostasis capacity (Meller and Shalgi, 2021). As organisms age, the efficiency of protein folding, trafficking, and degradation decreases, creating an environment that supports the accumulation of misfolded proteins such as Aβ, tau, α-synuclein, and TAR DNA-binding protein 43 (TDP43) (Wolozin and Ivanov, 2019). Major processes, including oxidative stress, mitochondrial dysfunction, and neuroinflammation, are connected to this malfunction of proteostasis mechanisms and can lead to cell death (Jellinger, 2010). The dysfunction of protein degradation pathways, such as the UPS and autophagy, contributes to the increase of misfolded proteins in neurodegenerative diseases, including AD (Jiang et al., 2025; Nixon and Rubinsztein, 2024; Nixon and Yang, 2011; Rao et al., 2015).

Lipid metabolism and cholesterol homeostasis play a significant role in AD pathogenesis. Disruption of these processes—including altered lipid composition and cholesterol transport—have been linked to AD pathology and disease progression. Such disturbances may exacerbate Aβ accumulation and tau phosphorylation, and contribute to neuroinflammation as well as myelin abnormalities (Ahmed et al., 2024; He et al., 2025; Kawade and Yamanaka, 2024; Mi et al., 2023). Importantly, restoring dysregulated lipid metabolism has been demonstrated to ameliorate AD-related pathologies (He et al., 2025; Litvinchuk et al., 2024).

One crucial modifying factor in AD is the fat-binding protein ApoE, which is central in transporting cholesterol and other lipids from astrocytes to neurons (Raulin et al., 2022; Windham and Cohen, 2024). Variants in the coding *APOE* gene have significant implications for AD and its treatment, especially in its late-onset sporadic form which occurs after the age of 65, where *APOE* is a key genetic risk factor (Islam et al., 2025). The three primary *APOE* allelic variants differ in two amino acid residues at positions 112 and 158 in the encoded protein ApoE — ε2 (Cys112; Cys158), ε3 (Cys112; Arg158), and ε4 (Arg112; Arg158) (Serrano-Pozo et al., 2021). Strong evidence from clinical and basic research indicates that the *APOE* ε4 allele is associated with an increased risk of AD (Corder et al., 1993), while the *APOE* ε2 allele is linked to a decreased risk compared to the more common *APOE* ε3 allele (Bu, 2009; Corder et al., 1994; Farrer et al., 1997). Mechanistically, *APOE* ε4 was implicated in disturbances of the lipid metabolism, primarily associated with impaired function in lipid and cholesterol efflux from astrocytes and neurons, leading to detrimental accumulation of lipid deposits (Lin et al., 2018; Raulin et al., 2022; Sienski et al., 2021). Notably, ApoE is known to accumulate in Aβ plaques and trigger tau hyperphosphorylation as well as its deposition (Hou et al., 2020; Namba et al., 1991; Therriault et al., 2020; Xia et al., 2024). Moreover, ApoE seems to play a protective role by mediating the removal of Aβ via receptor-mediated clearance and extracellular proteolytic machineries, while the ApoE ε4 presented impaired functionality in these pathways, contributing to disease-associated accumulation of extracellular plaques (Jiang et al., 2008; Kanekiyo et al., 2013; Van Acker et al., 2019).

The variations in ApoE, particularly the protective effects of the *APOE* ε2 allele and the risk associated with *APOE* ε4, present critical insights for AD research. Understanding these genetic factors can enhance diagnostic precision and guide the development of targeted therapies, offering significant potential for more effective treatment strategies (Hou et al., 2020; Namba et al., 1991; Therriault et al., 2020; Xia et al., 2024).

From the perspective of AD progression, all the factors mentioned above contribute and define the pathological stages of this disorder. In the asymptomatic phase, amyloid-beta (Aβ) plaques begin to accumulate, a process strongly influenced by lipid metabolism and ApoE function (Raulin et al., 2022). During the prodromal or mild cognitive impairment (MCI) phase, tau pathology emerges, and subtle cognitive deficits appear, with ongoing ApoE- and lipid-mediated effects on protein clearance, membrane composition, and synaptic function. In the dementia phase, widespread neuronal loss, synaptic dysfunction, and cognitive decline occur, with dysregulated ApoE and lipid homeostasis further exacerbating proteostasis impairment, inflammation, and neurodegeneration (Jack et al., 2010; Dubois et al., 2016; Zhang et al., 2024).

#### 2.1.2 Cerebral amyloid angiopathy (CAA)

CAA is one of the main causes of lobar intracerebral hemorrhage (ICH) in the elderly, causing 5–20% of spontaneous ICH in older adults (de Bruin et al., 2024). The prevalence of CAA increases significantly with age and is observed in approximately 80–90% of individuals with AD pathology (Yamada, 2015). CAA is characterized by vascular deposition of Aβ in the walls of small leptomeningeal arteries and cortical blood vessels (de Bruin et al., 2024) (Figure 1). Despite its close pathological overlap with AD, CAA shows distinct clinical features and may thus act as a pathological bridge linking cerebrovascular dysfunction with other neurodegenerative diseases (Cordonnier and van der Flier, 2011).

Structurally, CAA differs from AD in terms of the predominant Aβ isoforms involved. Unlike in AD, shorter Aβ fragments are more abundantly deposited in CAA. While Aβ42 is primarily deposited in parenchymal neuritic plaques in AD, CAA is characterized by the more abundant deposition of Aβ40. Moreover, several studies have demonstrated that shorter Aβ isoforms—such as Aβ37, Aβ38, and Aβ39—are also present in vascular deposits (Kakuda et al., 2017; Reinert et al., 2016). These more soluble isoforms are thought to follow perivascular drainage pathways, which may contribute to their selective vascular accumulation (Greenberg et al., 2020; van den Berg et al., 2024). Over time, Aβ accumulation leads to the loss of vascular smooth muscle cells (VSMCs). These contractile cells, believed to be of mesenchymal origin (Sinha et al., 2014), are located in the tunica media (middle layer) of small arteries and arterioles, where they play a crucial role in maintaining vessel tone, regulating cerebral blood flow, and preserving vascular integrity. In CAA, their gradual depletion—particularly in leptomeningeal and cortical arteries—weakens the vessel wall and increases its risk of rupture. Despite these changes, the precise trigger initiating peptide deposition remains unknown. However, it is widely believed that these deposits result from impaired Aβ clearance, rather than overproduction, in the vascular walls, ultimately compromising vessel integrity (Koemans et al., 2023; Qi and Ma, 2017).

Similar to observations in AD, proteostasis and proteolytic mechanisms critical for Aβ generation and clearance are disrupted in CAA (Krohn et al., 2011; Ma et al., 2010; Monro et al., 2002; Savar et al., 2024; Qi and Ma, 2017). Moreover, vascular Aβ accumulation appears to involve lipid components (de Oliveira et al., 2025) and autophagy, an essential mechanism that maintains cellular health and homeostasis by removing damaged proteins and organelles (Liu et al., 2023).

Autophagy is characterized by membrane structures that form the autophagosomes—double-membrane vesicles that engulf cellular material for degradation. The fusion of autophagosomes with lysosomes vesicles containing hydrolytic enzymes enables the breakdown of cellular components (He and Klionsky, 2009). Experimental studies suggest that activating autophagy may have beneficial effects in CAA (Ma et al., 2010). This is mediated by the transmembrane lipoprotein receptor-related protein 1 (LRP1), which plays a key role in Aβ uptake and lysosomal degradation (Bell et al., 2009; Cheung et al., 2014; Kanekiyo et al., 2012). Notably, LRP1 is also a major neuronal ApoE receptor and is involved in modulating Aβ pathology (Na et al., 2023; Shinohara et al., 2017; Strickland and Holtzman, 2019; Tachibana et al., 2019). Previous *in vitro* and *in vivo* studies have shown that the ApoE protein influences multiple aspects of Aβ pathology, including its accumulation, clearance, conformational state, and toxicity (Rannikmäe et al., 2013). The *APOE* ε4 allele is associated with an increased risk of CAA, as it impairs Aβ clearance from the brain and promotes vascular deposition. In contrast, the ε2 allele—although considered protective in AD—may increase the risk of vessel wall fragility in CAA due to Aβ accumulation, thus predisposing to ICH recurrence. Interestingly, individuals with the *APOE* ε2/ε4 genotype may experience a compounded pathological effect, with both enhanced Aβ deposition and increased vascular fragility, leading to a higher risk of early ICH recurrence (Greenberg et al., 2020; Yang et al., 2025).

These findings show that the *APOE* gene influences the development of CAA and could be important for diagnosis as well as potential treatment.

### 2.2 Inherited neurodegenerative disorders

Strictly hereditary neurodegenerative disorders, which account for approximately 10% of all cases, are genetically heterogeneous and involve mutations in genes such as *presenilin-1* (*PSEN1*) in familial AD, *leucine-rich repeat kinase 2* (*LRRK2*) and *parkin* (*PRKN*) in dominant or recessive forms of PD, and *C9orf72* in Fronto Temporal Dementia (FTD) and amyotrophic lateral sclerosis (ALS) (Dilliott et al., 2021; Pihlstrøm et al., 2017). A distinct group within inherited neurodegenerative diseases comprise the so-called polyglutamine (polyQ) disorders, which include the following nine conditions: spinobulbar muscular atrophy (SBMA), dentatorubral-pallidoluysian atrophy (DRPLA), Huntington's disease (HD), and six spinocerebellar ataxias (SCA1, SCA2, SCA3, SCA6, SCA7, and SCA17). All of these diseases follow an autosomal dominant pattern of transmission, except for SBMA, which is X-linked recessive (Orr and Zoghbi, 2007).

#### 2.2.1 Polyglutamine (polyQ) disorders

PolyQ diseases are characterized by a CAG trinucleotide expansion in the coding region of the affected gene, leading to an elongated polyQ tract in the translated protein. The length of the CAG repeat directly influences the age at onset, with longer CAG repeat expansions correlating with earlier disease onset and more severe manifestation of symptoms (Tandon et al., 2024). The polyQ expansion alters properties of the affected protein, induces its misfolding, and results ultimately in its accumulation in the form of neuronal intracellular aggregates (Figure 1). These aggregates may contain not only the entire protein or its polyQ stretch-containing fragments, but also additional components such as chaperones, ubiquitin, ubiquitin-binding proteins, proteasomal subunits, and other vital factors such as transcription-related proteins (Havel et al., 2009; Lieberman et al., 2019). Although the disease-causative proteins of all polyQ diseases are ubiquitously expressed across different cell types, aggregate formation and cell loss is restricted to neurons, while the affected brain region differs depending on the disease. Apart from the expanded polyQ stretch, the affected proteins lack sequence homology and participate in diverse cellular processes, including transcription regulation, RNA metabolism, protein homeostasis, and protein-protein interactions (Maiuri et al., 2017; Paulson et al., 2017). Despite differences in the affected neuronal subpopulations and the varied functions of the disease-causing proteins, polyQ diseases share several pathological features (Figure 1B), which may represent common targets for therapeutic development.

Below, we present a selected overview of spinocerebellar ataxias (SCAs) 2 and 3, along with HD, as representative polyQ disorders, and highlight their common targetable pathways, with a focus on the impact of ApoE and lipid metabolism on pathology and proteostasis.

#### 2.2.2 Spinocerebellar ataxia type 2 (SCA2)

SCA2 (OMIM: #183090) is caused by an expansion of a CAG tract in exon 1 of the *ATXN2* gene that encodes the protein ataxin-2 (Pulst, 1993). Repeat lengths up to 31 CAG repeats are considered normal alleles (de Castilhos et al., 2014; Gardiner et al., 2019), with a high prevalence of 22 CAG repeats, being found in 90.1% of the general population (Andrés et al., 2003), while expansions of 33 and above are considered as fully penetrant for SCA2. Repeat lengths around this threshold and CAA interruptions have been additionally associated with other neurodegenerative disorders, causing recessive SCA2 or representing a risk factor for ALS and PD (Charles et al., 2007; Elden et al., 2010; Gwinn-Hardy et al., 2000; Tojima et al., 2018). Expanded CAG repeats and consequently longer polyQ tracts in ataxin-2 lead to toxicity and neurodegeneration in the cerebellum and brainstem (Bunting et al., 2022). Clinical symptoms frequently observed are progressive ataxia and dysarthria, slow saccadic eye movements, and peripheral neuropathy (Pulst, 1993).

The known physiological role of wild-type ataxin-2 includes its posttranscriptional regulatory function in RNA metabolism and translation, and its involvement in cytoplasmic stress granules (Carmo-Silva et al., 2017; Costa et al., 2024). While the polyQ expansion in ataxin-2 likely alters its function, promoting toxicity and aggregation, the role of ataxin-2 aggregates—primarily due to their infrequent nuclear localization—was initially considered of minor pathological relevance. Notably, neuronal loss and intranuclear inclusions are not necessarily concomitant in SCA2 (Huynh et al., 2000; Koyano et al., 2014). However, the presence of cytoplasmic aggregated polyQ-expanded ataxin-2, visible as granular staining, has been shown to correlate with disease progression in the SCA2 patient brain (Koyano et al., 2014; Seidel et al., 2017). Dysregulation of autophagy has been observed in both SCA2 mouse models and patient-derived samples, indicating disease-related disruptions that may impair the toxic protein clearance and promote aggregation. Conversely, activation of this degradation pathway or its upstream regulators was found to ameliorate SCA2 pathology (Afonso et al., 2022; Liu et al., 2024; Marcelo et al., 2021; Paul et al., 2018; Wardman et al., 2020).

Ataxin-2 also plays a role in lipid metabolism. Knockout models revealed various perturbations, including deficits in lipid and cholesterol metabolism (Lastres-Becker et al., 2008). In the brains of SCA2 knock-in mice, reduced levels of myelin lipids and downregulation of enzymes essential for cholesterol biosynthesis were observed, accompanied by decreased levels of cholesterol precursor metabolites (Canet-Pons et al., 2021; Sen et al., 2019). Similarly, the primary cholesterol elimination product, 24S-hydroxycholesterol, was found to be reduced in the brains of SCA2 patients (Locci et al., 2023). However, little is known about the involvement of ApoE in SCA2, although some studies have reported increased ApoE protein or expression levels in patient blood or fibroblasts (Cornelius et al., 2017; Swarup et al., 2013).

#### 2.2.3 Spinocerebellar ataxia type 3 (SCA3)/Machado-Joseph disease (MJD)

SCA3, or Machado-Joseph disease (MJD) (OMIM: #109150), represents the second most common polyQ disease after HD, and the most common SCA worldwide (Durr, 2010; Gardiner et al., 2019; Klockgether et al., 2019). It is caused by a CAG repeat expansion in exon 10 of the *ATXN3* gene. Repeat lengths of 12 to 44 CAGs are found in non-affected individuals, while around 56 to 87 repeats are associated with clinical manifestation of the disease. For intermediate repeat lengths, incomplete penetrance of symptoms has been reported (McLoughlin et al., 2020). Symptoms include progressive cerebellar ataxia with motor deficiencies such as gait abnormalities, coordination problems, impaired balance or oculomotor impairments, but can also include parkinsonism, sleep disturbances or sensory damage (Rüb et al., 2013).

At the molecular level, the polyQ-expanded SCA3 disease-related protein ataxin-3 is abnormally folded and accumulates as intracellular protein aggregates, which works as a bait for other proteins, ultimately disrupting multiple cellular processes (Yang et al., 2014). Ataxin-3 is a deubiquitinase and mediates protein quality control pathways such as autophagy and the UPS, which are compromised in SCA3 and can be targeted for improving the molecular phenotype (Ashkenazi et al., 2017; Blount et al., 2014; Costa Mdo and Paulson, 2012; Menzies et al., 2010; Onofre et al., 2016; Pereira Sena et al., 2021). Apart from this function, ataxin-3 has also been associated with DNA damage repair (Gao et al., 2015; Pfeiffer et al., 2017) and transcriptional regulation (Li et al., 2002). Therefore, the ramifications of mutant ataxin-3 on protein homeostasis are not limited to aggregation and aberrant degradation of the actual disease protein but also impact the turnover of multiple other cellular proteins.

Studies on pathological dysfunctions in lipid and cholesterol metabolism in SCA3 remain limited but consistently highlight their significant impact, as demonstrated in various models and patient materials (Campos et al., 2022; Putka et al., 2025; Toonen et al., 2018). Notably, restoration of cholesterol levels by viral administration of cholesterol 24-hydroxylase (CYP46A1) in SCA3 mice activated autophagy, enhanced aggregate clearance, and concurrently alleviated both neuropathology and motor deficits (Nóbrega et al., 2019).

While the main factor contributing to age at onset is the length of the CAG repeat, genetic modifiers have been identified in SCA3 (de Mattos et al., 2019; Raposo et al., 2022; Weber et al., 2024), including the *APOE* genotype. It was demonstrated that carriers of the *APOE* ε2 allele present an earlier disease onset (Bettencourt et al., 2011a; Peng et al., 2014), although a later study did not come to this conclusion (Zhou et al., 2014). There is, however, evidence that the *APOE* ε4 allele is associated with better performance in language and visual memory in SCA3 patients, while being also associated with rather severe speech disturbances (Chen et al., 2025). A further case report of two SCA3 patients presenting parkinsonism identified a common ApoE genotype, namely *APOE* ε2/ε3 (Bettencourt et al., 2011b). Since *APOE* ε2 has previously been associated with PD (Huang et al., 2004; Jo et al., 2021; Pang et al., 2018), it is reasonable to suggest that *APOE* ε2 is linked to parkinsonism in SCA3. However, the exact repercussions of the *APOE* genotype on the molecular pathogenesis of SCA3, in particular in the proteostatic networks, have not been addressed yet.

#### 2.2.4 Huntington's disease (HD)

With an estimated global prevalence of 4.88 per 100,000 individuals, HD (OMIM: #143100) is the most common inherited neurodegenerative disease and polyQ disorder (Arrasate and Finkbeiner, 2012; Medina et al., 2022). An aberrant expansion of the glutamine-coding CAG repeat region in exon 1 of the *HTT* gene leads to disease onset, with full disease penetrance to be expected above 39 CAG repeats (Jiang et al., 2023). Although symptoms vary between affected patients, HD is typically characterized by progressive motor disability and cognitive decline, chorea, personality changes and mood disorders, speech difficulties, and impaired gait, balance, and coordination (Lieberman et al., 2019; Saudou and Humbert, 2016). Neuropathologically, HD involves early degeneration of striatal GABAergic medium spiny neurons, leading to striatal atrophy and cortical thinning years before symptom onset (Aylward et al., 2011; Ehrlich, 2012; Rosas et al., 2008; Ross and Tabrizi, 2011).

The precise molecular function of wild-type HTT protein is still unknown. However, it is suggested to be a multivalent structural scaffolding hub for proteins by mediating crucial intra- and inter-molecular protein interactions via its HEAT domains (Saudou and Humbert, 2016). The interplay of these interactors with HTT dictates its physiological role in vesicle trafficking and recycling, cell division, ciliogenesis, endocytosis, autophagy, and transcriptional regulation, with many of these pathways being compromised upon polyQ expansion (Ehrnhoefer et al., 2011; Saudou and Humbert, 2016).

In the nucleus and cytoplasm of HD brain neurons, large intracellular aggregates of polyQ-expanded HTT, termed inclusion bodies, were reported (Difiglia et al., 1997; Gutekunst et al., 1999). Although aggregates are generally believed to have detrimental effects on cell viability, a causative relation between aggregate formation and cell death has not yet been drawn. However, it is hypothesized that inclusion bodies may mediate their toxic function through their sequestration of important cellular proteins, such as transcription factors or UPS-components, essentially rendering them dysfunctional (Leverenz et al., 2007; Lutz and Peng, 2018; Riguet et al., 2021; Shahmoradian et al., 2019; Stewart and Radford, 2017).

Consequently, multiple studies have investigated ways to activate autophagy or the UPS to eliminate detrimental soluble or aggregated forms of the polyQ-expanded HTT (Bailus et al., 2021; Bhat et al., 2014; Ravikumar et al., 2004; Williams et al., 2008).

As the CAG repeat length explains only 50% of the total variance in age at HD onset, other factors, such as a perturbed lipid metabolism and, in particular, ApoE, have been proposed to contribute to HD progression and molecular pathogenesis (Figure 1B) (Block et al., 2010; Panas et al., 1999). Interestingly, clinical data on HD revealed that the *APOE* ε4 allele in patient carriers delayed the age at onset of HD by a mean difference of 13.6 years, compared to ε3/ε3 patients (Panas et al., 1999). Regardless of specific isoforms, a reduction of ApoE synthesis and secretion in HD astrocytes were found in various rodent models of HD (Valenza et al., 2010, 2015). As neurons in the adult brain are mostly dependent on astrocyte synthesis and efflux of cholesterol (Saher and Stumpf, 2015), increasing ApoE-mediated cholesterol efflux from astrocytes could potentially lessen cholesterol-dependent neuronal damage in HD (Valenza et al., 2010). Furthermore, gene-therapeutic delivery of CYP46A1 into the striatum of HD affected mice not only improves disease pathology and reduces mutant HTT aggregates, but also enhances cholesterol metabolism by upregulating the expression of cholesterogenic enzymes and ApoE (Boussicault et al., 2016; Kacher et al., 2019, 2022). Additionally, dysregulation of cholesterol metabolism in HD models has been shown to alter mitochondrial membrane (MM) fluidity, whereas administration of the neuroprotective cholesterol derivative olesoxime exerted restorative effects, potentially by enhancing MM cholesterol levels (Eckmann et al., 2014; Weber et al., 2019). HTT itself has been found to associate with lipids and undergo lipidation, and its interactions with cholesterol, lipids, or lipid membranes have been shown to influence its aggregation (Beasley et al., 2021; Lemarié et al., 2023; Stonebraker et al., 2023). These findings in HD models suggest a potentially broader link between lipid metabolism, ApoE, and proteostasis.

## 3 Potential points of intervention

Although still incurable, multiple therapeutic strategies have been explored for the sporadic and monogenic neurodegenerative disorders discussed in this review. These approaches include molecules acting on known dysregulated neuronal signaling pathways, small molecules targeting protein aggregation, antisense oligonucleotides or RNA-based therapies to reduce toxic protein expression, gene therapy strategies aimed at restoring normal protein function, and immunotherapies directed against extracellular aggregates (Zhang et al., 2024; de Sousa-Lourenço et al., 2024; Tenchov et al., 2024). Notably, a recent report from the pharmaceutical company UniQure announced a first-time slowdown in HD progression by 75% using a surgical, microRNA-based strategy aiming at lowering the mutant HTT protein (https://www.clinicaltrials.gov/study/NCT04120493). Despite promising preclinical and early clinical results, most of these interventions have yet to achieve clear disease-modifying effects in patients in a practicable manner.

Since protein aggregation is a hallmark across multiple sporadic and monogenic neurodegenerative disorders, current efforts focus on reducing these intra- or extracellular deposits or their sources. Strategies to activate often-compromised proteolytic systems, such as autophagy and the UPS, include genetic and pharmacologic manipulation of pathway-related genes, modification of their upstream regulators or effector elements, and the targeting of substrate proteins via posttranslational modifications (Dantuma and Bott, 2014; Le Guerroué and Youle, 2021; Nixon and Rubinsztein, 2024). However, thinking beyond conventional approaches by including additional dysregulated, targetable pathways can broaden the strategic range. This may enable the development of novel, potentially more feasible interventions, that not only restore proteostasis but also address other pathologically impaired molecular mechanisms. Here, observations in monogenic diseases with a clearer molecular etiology can offer an advantage in assessing the robustness of identified points of action. One such compromised, broader pathway may be lipid metabolism—particularly the involvement of one of its key components, ApoE, across various neurodegenerative disorders (Estes et al., 2021; Fernández-Calle et al., 2022). Numerous studies have demonstrated that lipid metabolism and autophagy modulate each other reciprocally, a relationship that becomes especially apparent when considering that autophagosomes and lysosomes are lipid-membrane vesicles, and that one autophagy protein, LC3, requires lipidation for activation (Jarocki et al., 2024; Xie et al., 2020). The regulatory effects of lipids on the UPS are less well understood. However, ubiquitination and the UPS play important roles in regulating lipid biosynthesis and turnover (Jiang and Song, 2014; Loix et al., 2024). Consequently, direct or indirect enhancement of autophagy may alleviate the burden on an overwhelmed UPS by restoring proteostasis—while simultaneously exerting beneficial effects on UPS-controlled lipid homeostasis. Exemplary studies conducted in SCA3, HD and, to a lesser extent, SCA2 models convincingly demonstrated that modifying cholesterol biosynthesis can ameliorate disease symptoms *in vivo* by enhancing autophagy and the UPS (Kacher et al., 2019; Nóbrega et al., 2019) (Table 1). Similarly beneficial effects were observed with small molecules that counteracted the autophagy-suppressing influence of the ApoE ε4 allele (Balasubramaniam et al., 2024; Parcon et al., 2018).

**Table 1:** Selected pathway implications, ApoE involvement, and experimental therapeutic strategies targeting lipid homeostasis in sporadic and monogenic neurodegenerative disorders.

**Disease (etiological form)**	**Impaired pathways of interest**			**ApoE as GM**	**LH-targeting therapeutic strategy for enhancing proteostasis**
	**Proteostasis**	**LH**	**ApoE**		
AD (sporadic)	X	X	X	Yes	Rescue of lipid metabolism He et al., 2025; Litvinchuk et al., 2024
CAA (sporadic)	X		(X)	Yes	N/A
SCA2 (monogenic)	X	X	X	N/A	N/A
SCA3 (monogenic)	X		(X)	Yes	Restoration of cholesterol levels via CYP46A1 Nóbrega et al., 2019
HD (monogenic)	X	X	X	Yes	Restoration of cholesterol levels via CYP46A1 Boussicault et al., 2016; Kacher et al., 2019, 2022 Olesoxime administration Eckmann et al., 2014; Weber et al., 2019

AD, Alzheimer's disease; CAA, cerebral amyloid angiopathy; SCA2, spinocerebellar ataxia type 2; SCA3, spinocerebellar ataxia type 3; HD, Huntington's disease; GM, genetic modifier; LH, lipid homeostasis; N/A, not available; X, implicated; (X), limited implication.

Pursuing comparable strategies across different neurodegenerative diseases and rigorously evaluating their effects and involved pathways will be essential to assess their potential as targets for unified therapeutic approaches.

## 4 Conclusion

The complexity and heterogeneity of the pathways affected in neurodegenerative disorders present major challenges for therapeutic development, contributing to the ongoing lack of effective treatments for many of these diseases. While pathways are well elaborated for AD, monogenic models such as polyQ disorders represent a better paradigm for analyzing molecular pathogenesis, since they are likely less challenging to be reproduced in cell and animal models.

The identification of common molecular targets—such as ApoE—across interconnected pathways like lipid homeostasis and proteostasis, and across multiple disorders (Table 1), has renewed interest in the search for effective therapies. A key challenge in this context will be to unravel the distinct contributions of ApoE's different variants to the disruption or maintenance of proteostasis, specially in monogenic neurodegenerative disorders—where the role of lipid metabolism and ApoE still remain underinvestigated. Once clarified, ApoE may emerge as a central player and promising target for therapeutic intervention in neurodegeneration.
